# Biodegradable Metal Complex-Gated Organosilica for Dually Enhanced Chemodynamic Therapy through GSH Depletions and NIR Light-Triggered Photothermal Effects

**DOI:** 10.3390/molecules29051177

**Published:** 2024-03-06

**Authors:** Lin Kong, Jian Li, Yunxiu Zhang, Jian Wang, Ke Liang, Xiaokuang Xue, Tiejin Chen, Yongliang Hao, Haohui Ren, Pengfei Wang, Jiechao Ge

**Affiliations:** 1 Key Laboratory of Photochemical Conversion and Optoelectronic Materials and CityU-CAS Joint Laboratory of Functional Materials and Devices, Technical Institute of Physics and Chemistry, Chinese Academy of Sciences, Beijing 100049, China; konglin20@mails.ucas.ac.cn (L.K.); zhangyunxiu114@mails.ucas.ac.cn (Y.Z.); wangjian18@mails.ucas.edu.cn (J.W.); liangke18@mails.ucas.ac.cn (K.L.); xuexiaokuang19@mails.ucas.ac.cn (X.X.); chentiejin20@mails.ucas.ac.cn (T.C.); haoyongliang22@mails.ucas.ac.cn (Y.H.); lindaroxanneren@mail.ipc.ac.cn (H.R.); wangpf@mail.ipc.ac.cn (P.W.); 2 School of Future Technology, University of Chinese Academy of Sciences, Beijing 100049, China

**Keywords:** biodegradable organosilica, chemodynamic therapy, GSH depletion, photothermal effect enhancement

## Abstract

Hollow silica spheres have been widely studied for drug delivery because of their excellent biosecurity and high porosity. However, difficulties with degradation in the tumor microenvironment (TME) and premature leaking during drug delivery limit their clinical applications. To alleviate these problems, herein, hollow organosilica spheres (HOS) were initially prepared using a “selective etching strategy” and loaded with a photothermal drug: new indocyanine green (IR820). Then, the Cu^2+^–tannic acid complex (Cu-TA) was deposited on the surface of the HOS, and a new nanoplatform named HOS@IR820@Cu-TA (HICT) was finally obtained. The deposition of Cu-TA can gate the pores of HOS completely to prevent the leakage of IR820 and significantly enhance the loading capacity of HOS. Once in the mildly acidic TME, the HOS and outer Cu-TA decompose quickly in response, resulting in the release of Cu^2+^ and IR820. The released Cu^2+^ can react with the endogenous glutathione (GSH) to consume it and produce Cu^+^, leading to the enhanced production of highly toxic ·OH through a Fenton-like reaction due to the overexpressed H_2_O_2_ in the TME. Meanwhile, the ·OH generation was remarkably enhanced by the NIR light-responsive photothermal effect of IR820. These collective properties of HICT enable it to be a smart nanomedicine for dually enhanced chemodynamic therapy through GSH depletions and NIR light-triggered photothermal effects.

## 1. Introduction

Malignant tumors seriously endanger global public health. Due to the heterogeneity of the TME [1,2,3], the efficacy of traditional treatment is often unsatisfactory. In addition, traditional treatment has obvious side effects on patients, and the tumor is prone to relapse, which greatly increases the pain of patients [3,4]. Thus, it is essential to explore new treatment methods. As a new emerging technology, phototherapy has shown great potential in the field of cancer treatment due to its non-invasiveness, high biosafety, and effect of a low recurrence rate [5,6]. The basic principle is that, under a specific wavelength of laser irradiation, the phototherapy regents can produce toxic ^1^O_2_ or convert light into heat [7,8], to disrupt the growth process of cancer cells. However, a single treatment modality often fails to cure the tumor; therefore, it is necessary to combine two or more treatment modalities to overcome the challenge of the poor efficacy resulting from monotherapy [9,10].

Chemodynamic therapy (CDT), which is highly logical and selective, catalyzes the conversion of overexpressed hydrogen peroxide (H_2_O_2_, 50–100 μM) at tumor sites into highly toxic hydroxyl radicals (·OH) through a Fenton-like reaction [11,12]. However, due to the enriched glutathione (GSH, ~10 mM) in the TME, the obtained ·OH can be partly consumed before taking effect [13]. Furthermore, the hypoxic TME with a low H_2_O_2_ concentration (50–100 μM) limits ·OH production, which becomes another barrier for CDT [14]. As a matter of fact, the TME certainly limits the effectiveness of the therapy, but it also paves the way for a specific treatment [15,16]. Thus, it is urgent that we develop a multi-responsive nanoplatform for multimodal therapy, which can enhance the efficiency of CDT by exploiting the properties of the TME, while simultaneously avoiding damage to the normal cells. Based on this, several strategies have been exploited with promising therapeutic results: (1) the design of nanoplatforms with the capacity for GSH-depletion to amplify the oxidative stress of the TME [17,18]; (2) the development of nanoplatforms to enhance the catalytic performance using an external stimulus (e.g., heat, light, and microwave) [19,20]; and (3) the construction of nanoplatforms with multi-catalytic capabilities [21,22]. Despite the continuous progress of nanotechnology, with a great deal of multi-responsive nanoplatforms having been developed, designing and synthesizing nanodrug delivery systems responsive to the TME remains challenging [18].

Hollow silica spheres have attracted much interest in the field of photosensitizer delivery due to their excellent biosecurity, high specific surface area, and porosity [23,24]. The traditional inorganic silica spheres cannot be degraded in the TME due to their surface chemical bond of -Si-O-Si, which hinders their clinical application [25,26]. The introduction of disulfide bonds (-S-S-) on the surface of traditional inorganic silica spheres enables them to be degraded by overexpressed GSH in the TME, which significantly improves their biodegradability [27,28,29]. However, premature leakage and absorption by normal tissue frequently occur during the process of drug delivery [30]. In order to solve these issues, an effective method is to grow a layer of pore-sealing reagent on the surface of the silica spheres [31,32]. A pore-sealing reagent with both a tumor treatment and biodegradation ability was selected, which can not only plug the silica sphere pore channel but also enhance the therapeutic efficiency, and it will not cause additional biological toxicity [31].

A metal polyphenol complex is a better choice of hollow organosilica sphere-sealing reagent. Firstly, metal polyphenol complexes degrade in the weakly acidic pH of the TME, showing good biocompatibility [18]. Furthermore, higher valence metal ions coordinating with polyphenol compounds can react with the overexpressed GSH in the TME to form lower valence metal ions, which can then further react with the overexpressed H_2_O_2_ in a weakly acidic environment to produce the more toxic reactive oxygen species ·OH [20,22]. In addition, the polyphenol compounds can accelerate the conversion of high metal ions to low metal ions under weakly acidic conditions, which can improve the efficiency of the Fenton reaction. Therefore, the hole-sealing design of hollow organosilica spheres using a metal polyphenol complex can avoid the leaking of the photosensitizer in advance, as well as the absorption by normal tissues of human body. In addition, it can also allow for the material to have the function of tumor-specific treatment and make up for the insufficient efficacy of the single phototherapy method [14,17,33].

**Scheme 1:**
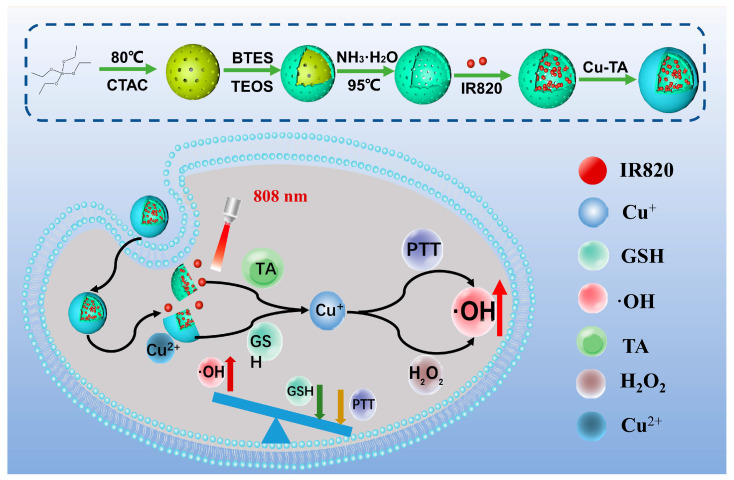
Schematic diagram of HICT and its therapeutic mechanism.

In this work, biodegradable metal complex-gated organosilica spheres were prepared for dually enhanced CDT through GSH depletions and NIR light-triggered photothermal effects. First, the inorganic/organic hybrid silica sphere HS@HOS was obtained through growth on the outside of the inorganic silica sphere. Following this, the inorganic silica spheres were etched inside with hot ammonia water to prepare the hollow organosilica sphere (HOS) carriers [34]. After loading the photothermal agent, new indocyanine green (IR820), into the HOS, tannic acid was deposited on the outside of the HOS. Then, after adding CuCl_2_ solution and adjusting the solution acidity, the copper–tannic acid complex (Cu-TA) grew in the outer layer of the HOS through the coordination reaction between metal ions and polyphenol compounds, and the assembled HOS@IR820@Cu-TA (HICT) was obtained. On the one hand, the introduction of the Cu-TA complex blocked the pores of the HOS and solved the problem of the IR820 leaking in advance during delivery and being absorbed by normal tissues. On the other hand, it endowed the material with chemodynamic properties, so that it could exert specific therapeutic effects in the TME and be degraded by the TME, ensuring the biological safety of the material. The HICT assembly can effectively generate heat under 808 nm laser irradiation, which can be used to kill tumor cells. In addition, Cu^2+^ can consume GSH in cancer cells to produce Cu^+^, which will react with overexpressed H_2_O_2_ to produce ·OH, further increasing the efficacy of the tumor treatment (Scheme 1). This work not only realized an efficient CDT with GSH consumption and photothermal enhancement but also provided a new idea for designing a smart nanoplatform to achieve efficient drug delivery.

## 2. Results and Discussion

### 2.1. Preparation of Hollow Organosilica Spheres Loaded with IR820 and Deposited Cu-TA (HICT)

**Figure 1:**
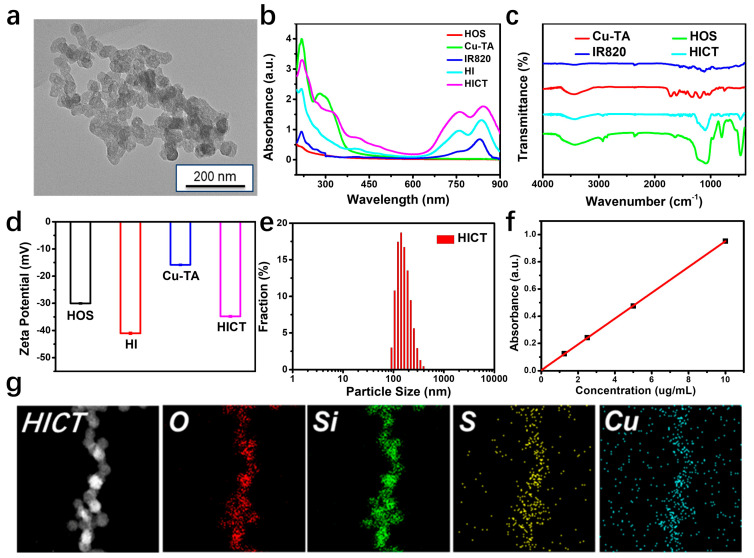
(**a**) Transmission electron microscopy of HICT; (**b**) UV–Vis absorption comparison of HOS, Cu-TA, IR820, HI (IR820 loaded HOS), and HICT. All of these were dispersed in pure water (pH 7.0), and the concentrations of IR820, HI, and HICT were 6, 60, and 200 μg/mL, respectively. (**c**) The FTIR spectra of the HOS, Cu-TA, IR820, HI (IR820 loaded HOS), and HICT. (**d**) Zeta potential of HICT and its components. (**e**) DLS particle size test chart of HICT. (**f**) Standard curve of the aqueous solution of IR820. (**g**) Element mapping image of the HICT assembly.

Hollow organosilica spheres (HOS) were synthesized by an “ammonia-selective etching strategy”. First, inorganic silica spheres (HS) were obtained by rapid hydrolysis of tetraethyl orthosilicate (TEOS) with cetyltrimethylammonium chloride (CTAC) as the pore-forming agent. After that, the organic silica source bis[3-(triethoxysilyl)propyl] tetrasulfide (BTPT) was deposited on the surface of the HS to yield HS@HOS. The HOS with a large surface area and aperture distribution could be prepared by using hot ammonia to selective etch away the HS core of the HS@HOS due to the weak resistance of HS to ammonia [35]. The transmission electron microscopy (TEM) of the HS@HOS (Figure S1a,b, Supporting Information) and HOS (Figure S1d,e) clearly showed a spherical morphology with an average diameter of 73.7 nm and 75.6 nm, respectively, revealed from the DLS results, as shown in Figure S1c,f. After loading the IR820 inside the HOS, the shell of the Cu-TA was deposited on the HOS surface through the coordination reaction between the natural polyphenol tannic acid (TA) as an organic ligand and Cu^2+^ to obtain hollow organosilica spheres loaded with IR820 and deposited Cu-TA (HICT). The TEM image clearly showed that the HICT possessed a spherical structure but with an uneven outer layer and a disappearing hollow structure compared to the HOS (Figure 1a). The absorption spectrum results showed that the HICT retained both the absorption peaks of IR820 at 700–900 nm and the absorption peak of Cu-TA at 250 nm (Figure 1b). Furthermore, Fourier-transform infrared (FTIR) spectroscopy was performed, as shown in Figure 1c. The stretching vibration peak (1090 cm^−1^) and bending vibration absorption (450 cm^−1^) of the Si-O were observed both in the HOS and HICT. The characteristic absorption peaks of 1550 cm^−1^ and 1725 cm^−1^, corresponding to the stretching vibration of the C=C and C=O bonds from TA, respectively, were observed in the Cu-TA and HICT. These typical peaks indicated the co-existence of Cu-TA and HOS in the HICT. As exhibited in Figure 1d, the zeta potentials of the synthesized HI and Cu-TA were −43 mV and −17 mV, respectively, while the zeta potential of the HICT changed to −36 mV, further confirming the successful preparation of the HICT. The hydrodynamic diameter of the HICT from the DLS measurement was about 165.6 nm (Figure 1e), which was suited to ingestion by tumor cells. In order to test the load capacity of the HOS on IR820, the standard curve of IR820 was drawn (Figure 1f), and the load capacity of the IR820 on HOS was calculated as 24.2%. The energy-dispersive spectroscopy (EDS) element mapping results and the XPS full spectrum of HICT indicated that the HICT was composed of O, Si, S, and Cu elements, consistent with the above results (Figure 1g and Figure S2).

Generally, when porous silica spheres deliver drugs, the drugs often leak before reaching the tumor site and are absorbed by normal tissues [36]. Herein, the Cu-TA complex was used to gate the pores of the HOS, and the effect was tested. As shown in Figure S3, after the Cu-TA growth on the surface of the HOS, the specific surface area was reduced from 362.4 m^2^/g to 197.0 m^2^/g, and the average pore size of the HOS originally decreased from 3.82 nm to 1.43 nm, which indicated that deposition of Cu-TA on the surface of silica spheres effectively blocked the pore channels of the HOS [31]. This presented a new method to overcome the challenge of avoiding the leakage of the porous silica spheres during drug delivery.

### 2.2. Photothermal Properties of HICT

**Figure 2:**
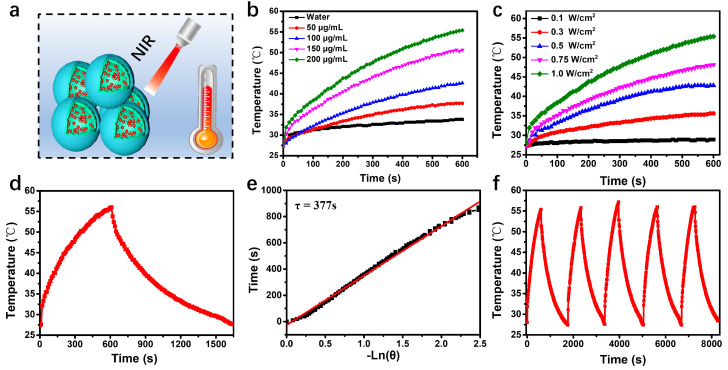
(**a**) Diagram of the photothermal conversion of HICT; (**b**) heating curves of different concentrations of the HICT solution within 10 min (808 nm laser, 1.0 W/cm^2^); (**c**) heating curves of the 200 μg/mL HICT solution within 10 min of irradiation by 808 nm lasers with different powers; (**d**) single irradiating and cooling curve of the 200 μg/mL HICT solution; (**e**) the linear fitting of the negative logarithm of temperature change (−ln(θ)) by the cooling phase time (t) of the 200 μg/mL HICT solution; (**f**) curve showing five cycles of irradiating and cooling of the HICT solution.

Since IR820 is an excellent photothermal reagent, and it was successfully loaded into the HOS, the temperature of the HICT assembly rose significantly after irradiation (Figure 2a). The photothermal properties of the HICT were tested, and the results indicated that the photothermal properties of HICT were positively correlated with the HICT concentration (Figure 2b). When the 808 nm laser was used to irradiate different concentrations of the HICT solution, the solution temperature significantly increased. The temperature of the 200 μg/mL HICT solution increased to 55.5 °C after 10 min irradiation, while the temperature of the water did not obviously change, demonstrating that the temperature increases in the solution mainly came from the conversion of the light energy absorbed by the HICT into heat energy. In addition, it can be seen that the photothermal performance of the HICT assembly was also dependent on the laser power (Figure 2c). When the 808 nm laser power increased, the HICT solution temperature range increased from 1.7 °C to 27.4 °C. Through fitting the single heating and cooling curve, the photothermal conversion coefficient τ was calculated to be 377 s, and the photothermal conversion efficiency (PCE) of HICT was 24.7% (Figure 2d,e) [37]. Finally, the photothermal stability of the HICT assembly was tested (Figure 2f). The HICT solution of 200 μg/mL was irradiated for 10 min using an 808 nm laser of 1.0 W/cm^2^, after which it was cooled to ambient temperature. The results showed that after five cycles, the maximum temperature that the HICT could reach did not change significantly, indicating that the HICT assembly had excellent photothermal stability and had potential as a photothermal reagent for application in tumor therapy.

### 2.3. Characterization of the Chemodynamic Performance

**Figure 3:**
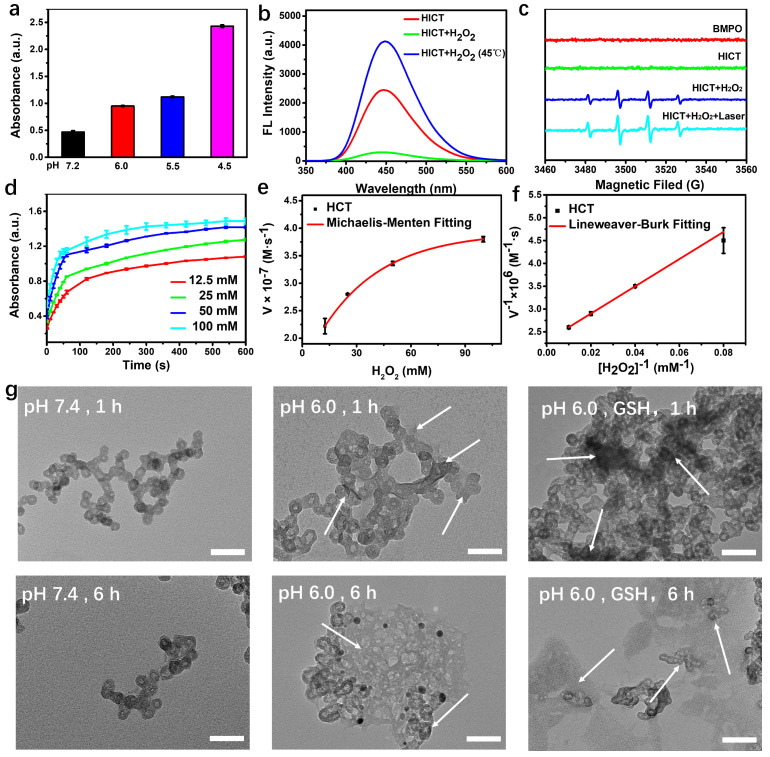
(**a**) The absorbance value of the oxidized TMB at different pHs. (**b**) Fluorescence spectra of NaTA under different treatments. (**c**) ESR spectra for DMPO-OH detection of different HICT-related treatments. (**d**) The absorption changes in oxidized TMB after HCT treatment with different concentrations of H_2_O_2_. (**e**) The Michaelis–Menten kinetics and (**f**) Lineweaver–Burk plot of HCT. (**g**) The morphology of HICT under different pH and GSH concentrations; the arrow points to the area where the responsive cleavage of HICT is most evident. Scale bar: 200 μm.

The presence of Cu^2+^ in the Cu-TA modified on the surface of HOS can react with the reducing substance GSH to convert it to GSSG. The DTNB was used to investigate the properties of the GSH consumed by the Cu-TA [31]. To avoid the influence of IR820 on the absorption spectrum measurement in the system, the GSH consumption properties of the Cu-TA modified on the HOS surface were tested using no loaded IR820 (named HCT). As shown in Figure S4, as the concentration of the HCT solution rose, the absorbance peak of the TNB decreased, suggesting the GSH was consumed by HCT. In addition, some studies have shown that tannic acid can accelerate the conversion of the Cu^2+^ to a Cu^+^ ion under acidic conditions, which can react with H_2_O_2_ to initiate a Fenton-like reaction. Thus, the TMB was used to detect the generated ·OH. With the increase in the acidity, the ability of the HCT to produce ·OH also increased (Figure 3a). The principle of NaTA as an ·OH-specific fluorescence probe is that it generates a strong fluorescence signal at a 310 nm wavelength after oxidation by ·OH, according to which the generation of ·OH can be detected [17]. The results showed that when HICT and NaTA were incubated together under acidic conditions, the system could only collect weak fluorescence signals, indicating that there was basically no ·OH formation at this condition. When H_2_O_2_ was added to the system, a strong fluorescence signal was generated, and the signal was enhanced when incubated at 45 °C (Figure 3b). The above results indicated that HICT can react with H_2_O_2_ to produce ·OH under acidic conditions, and the thermal effect can enhance the production of ·OH; the results of the ESR spectra further confirmed this conclusion (Figure 3c). In addition, to evaluate the catalytic activity of HCT, the Vmax (maximal reaction velocity) and Km (Michaelis constant) were calculated to be 4.3 × 10^−7^ M s^−1^ and 12.98 mM, respectively, by a typical Michaelis–Menten steady-state kinetic assay (Figure 3e,f). These results demonstrated that the reaction between the Cu^+^ generated from HCT and H_2_O_2_ conformed to steady-state dynamics [11]. To evaluate the biodegradability of the HICT, the morphology of the HICT at different pH and GSH concentrations was studied. As shown in Figure 3g, the morphology of the HICT did not change significantly under a simulated healthy physiological environment. However, in a weakly acidic environment (pH 6.0), the morphology of the HICT gradually collapsed as time passed, and the presence of GSH accelerated the occurrence of this phenomenon. These results indicated that the HICT assembly would be responsive to degradation in the TME and had good biosafety because the Cu-TA shell and disulfide bonds in the HOS gradually disassembled in the TME due to the protonation of tannic acid and the reducibility of the GSH.

### 2.4. In Vitro Anticancer Activity

**Figure 4:**
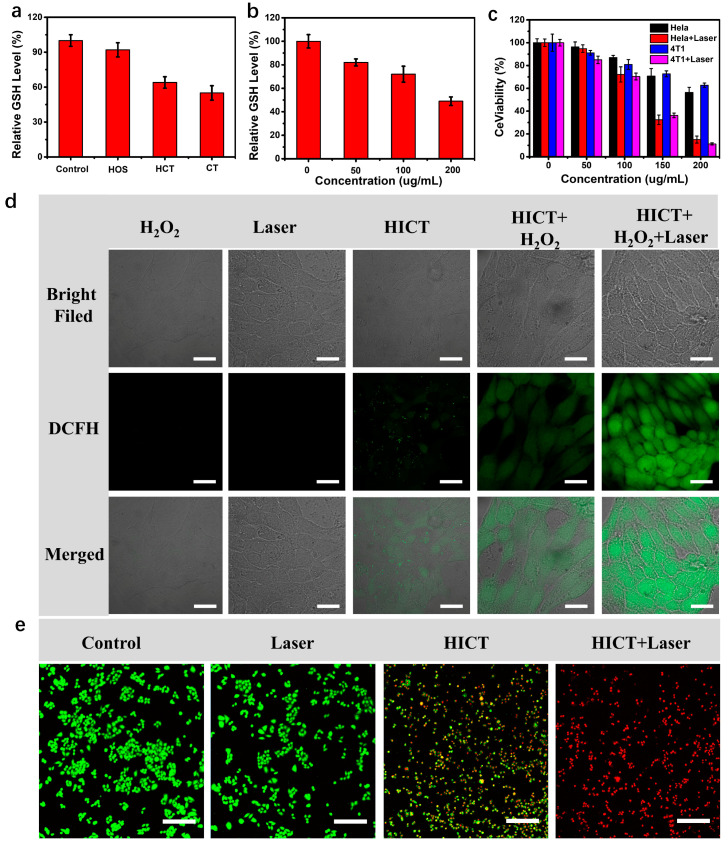
(**a**,**b**) Quantification analysis of GSH in 4T1 cells after different treatments. (**c**) Cytotoxicity of HeLa cells and 4T1 cells by MTT assays. (**d**) Fluorescence images of intracellular reactive oxygen species detected by DCFH-DA under different formulation treatments (H_2_O_2_, Laser, HICT, HICT + H_2_O_2_, HICT + H_2_O_2_ + Laser). (**e**) Fluorescence images of Calcein-AM and PI co-stained assays. Scale bar: 50 μm.

Due to the degradability of the HICT in the simulated TME, the biosafety of HICT was first determined. The results demonstrated that over 90% of the HU-EVC cells and MCF-10A cells were still alive after treatment with 200 μg/mL of HOS or HICT for 24 h (Figure S5), which indicated that the HICT had no obvious biological toxicity. Then, rhodamine B was loaded into the HOS (named RhB@HICT) to evaluate its uptake by tumor cells. The CLSM results showed that the fluorescence intensity reached the maximum at the eighth hour; so, this time was selected as the time point for the photothermal treatment (Figure S6). Since the released Cu^2+^ from the degradation of HICT could react with GSH, it was necessary to investigate the intracellular residual GSH concentration (Figure 4a). In comparison with the HOS, both HICT and Cu-TA (CT) could significantly decrease the intracellular GSH level, which is due to the reaction of the Cu^2+^ in the Cu-TA component with GSH. In addition, Figure 4b shows that the ability of HICT to consume GSH is proportional to its concentration. Considering the above experimental results, the Cu^+^ generated from the GSH-reduced Cu^2+^ in the Cu-TA component can react with H_2_O_2_ to produce highly toxic ·OH; this hypothesis was confirmed through the DCFH fluorescence probe to detect intracellular reactive oxygen species (ROS). HeLa cells treated with HICT showed only weak green fluorescence, and the fluorescence intensity increased significantly after the addition of H_2_O_2_, indicating that the level of ROS was upregulated. Moreover, the fluorescence intensity was the highest after laser irradiation (Figure 4d). All the results demonstrated that the photothermal effect could also enhance the production of ·OH at the cellular level, indicating that HICT has the potential to be used in photothermal-enhanced chemodynamic therapy. Thus, we investigated the anticancer activity of HICT in vitro using MTT assays. The viability of the HeLa cells and 4T1 cells treated with HICT alone reached 56.4% and 62.7%, approximately. However, the activity of the HeLa cells and 4T1 cells treated with the HICT under 808 nm laser irradiation (1.0 W/cm^2^, 10 min) decreased significantly, and their viability was only 15.07% and 11.23%, respectively (Figure 4c). To further demonstrate the anticancer capabilities of HICT, the Calcein-AM and PI co-staining experiment was conducted. The control and laser group showed almost green fluorescent signals, illustrating that laser irradiation alone could not kill cancer cells. The HeLa cells treated with HICT showed faint red fluorescence due to the chemodynamic effect; however, the HICT and laser co-treatment showed a strong red fluorescent signal (Figure 4e). These results indicate that the GSH-depleted and NIR photothermal dually enhanced chemodynamic therapy of HICT could achieve outstanding therapeutic results, and it offers a promising candidate for cancer treatment.

### 2.5. In Vivo Synergistic Anticancer Activity

**Figure 5:**
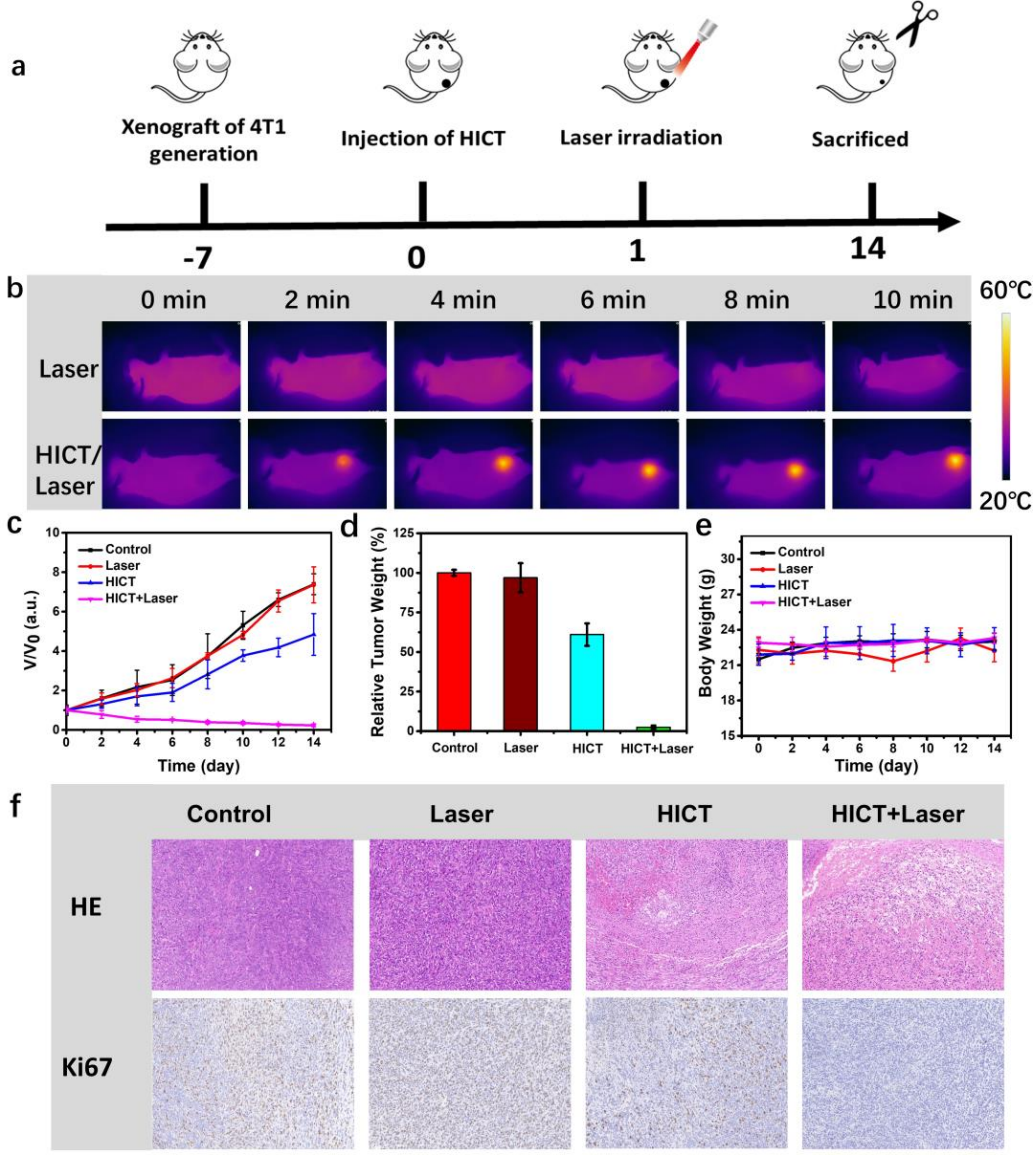
(**a**) Establishment and treatment schematic diagram of 4T1 tumor-bearing nude mice model. (**b**) Temperature of tumor tissue of mice during treatment. (**c**) Tumor volume, (**d**) relative tumor weight, and (**e**) weight changes in mice during treatment process. (**f**) H&E staining and Ki67 immunohistochemical staining under different treatments.

Inspired by the excellent results in the cell experiments of HICT, in vivo experiments were further conducted in 4T1 tumor-bearing mice. The schematic diagram of the in vivo experiments is shown in Figure 5a, and the 4T1 tumor-bearing mice were randomly divided into four groups: Control, Laser, HICT, HICT + Laser. The mice in the Laser and HICT + Laser groups were irradiated at 12 h post injection, and the temperature variations of the tumor tissues were recorded. Unlike the “Laser” group, the tumor temperature in the “HICT + Laser” groups remarkably increased to 56.9 °C after irradiation for 10 min, which was enough to kill tumor cells (Figure 5b). As shown in Figure 5c,d, the Control and Laser groups had almost no therapeutic effect, while a weakly therapeutic effect was achieved in the HICT group because of the CDT. Interestingly, the “HICT + Laser” group (808 nm, 1.0 W/cm^2^, 10 min) exhibited complete inhibition of tumor growth, originating from the GSH-depleted and NIR photothermal dually enhanced chemodynamic therapy. There was no significant weight loss in all mice, indicating that these treatments had good biological compatibility (Figure 5e). In addition, the pathological changes in the tumor tissues were evaluated by histological analysis. The H&E staining results indicated, different from the other three groups, that most of the tumor tissue in the “HICT + Laser” group was necrotic and damaged. In addition, the “HICT + Laser” group had the fewest Ki67-positive cells, indicating a significant inhibition of cancer cell proliferation. In addition, there were no obvious pathological abnormalities in the H&E staining of major organs in all groups, indicating that the biocompatibility of each treatment method was excellent (Figure S7) [38].

## 3. Materials and Methods

### 3.1. Materials

Octadecyl trimethyl ammonium chloride (CTAC), tetraethyl orthosilicate (TEOS), Bis [3-(triethoxysilyl) propyl] tetrasulfide (BTPT), tannic acid (TA), tetramethyl benzidine (TMB), and new indocyanine green (IR820) were purchased from Innochem Technology Corporation (Beijing, China). Triethanolamine (TEA), ammonium hydroxide (NH_3_·H_2_O), cupric chloride pentahydrate (CuCl_2_·H_2_O), glutathione (GSH), and dithio-dinitrobenzoic acid (DTNB) were purchased from Energy Chemical Corporation (Shanghai, China). Calcein-AM and PI were purchased from Beyotime Biotechnology (Nantong, China). Phosphate Buffer Solution, Fetal Bovine Serum, DMEM medium, penicillin, and streptomycin were purchased from Beijing Holide Technology Co., Ltd. (Beijing, China). All cells were cultured at 37 °C with 5% CO_2_.

### 3.2. Preparation of the Hollow Organosilica Spheres Loaded with IR820 and Deposited Cu-TA (HICT)

First, 4 g of CTAC and 0.2 g of TEA was dissolved in 40 mL of ultrapure water and stirred for 30 min. Then, we transferred the system to an oil bath at 80 °C, 2 mL of TEOS was added to the system, and the mixture was stirred for 4 h. The resultant HS@HOS was purified with ethanol three times, and the residual CTAC was extracted with acid ethanol (10% hydrochloric acid). The as-synthesized HS@HOS was selectively etched by NH_3_·H_2_O and washed with water to obtain HOS; then, the IR820 was loaded into the HOS. After that, 100 μL tannic acid (40 mg/mL) solution was added, it was eddy shocked for 5 min, the pH of the system was adjusted to 7.4 using HEPES buffer solution, and the reaction was continued for 10 min. Finally, the resultant HICT products were purified with water.

### 3.3. Measurement of the HICT Assembly

The DLS and zeta potential of the HICT nanoparticles in water were determined by a Malvern instrument Zeta Nano (Malvern Panalytical, Malvern, PA, USA). Transmission electron microscopy (TEM, Hitachi HT 7700, Hitachi, Tokyo, Japan) was used to observe the morphology of the HICT. The chemical structures were analyzed with XPS, and the specific surface area was determined by BET.

### 3.4. Detection of GSH Consumption

HCT (200 μg/mL) and GSH (1 mM) were dispersed in PBS buffer (pH 6.0) and then incubated for 2 h at 37 °C, followed by centrifugation (18,000 r/min, 10 min). Then, we added the DTNB solution into the supernatant, and the absorbance at 412 nm was recorded.

### 3.5. Measurement of ·OH Generation

The absorption peak at 650 nm after the oxidation of tetramethylbenzidine (TMB) by ·OH was used to characterize the generation of ·OH. Meanwhile, to avoid the influence of IR820 on the test system, hollow silica spheres without loading IR820 were used for testing, and the assembly modified with the metal complex Cu-TA, namely HCT, was used for testing. The TMB solution was prepared with ethanol at a concentration of 10 mM and diluted with PBS buffers at a pH of 7.2, 6.0, 5.5, and 4.5, respectively, so that the concentration was fixed at 1 mM. Then, H_2_O_2_ with a concentration of 10 mM was added to the system, and the HCT assembly was added. After 12 h of reaction, the absorption value change of the whole system at 650 nm was tested. The ·OH specific fluorescent probe NaTA and ESR detection were used to further verify the production of ·OH.

### 3.6. Photothermal Effect Evaluation

The photothermal effects of the HICT were evaluated under 808 nm laser irradiation. HICT solutions at various concentrations or fixed-concentration HICT suspensions (200 μg/mL) were illuminated by an 808 nm laser with different power densities, and the temperature changes were recorded. The HICT solution was repeatedly irradiated for five cycles to further investigate the photothermal stability.

### 3.7. Intracellular GSH Consumption Detection

The 4T1 cells were treated with (1) Control, (2) HOS, (3) HCT, and (4) Cu-TA and incubated for 24 h. Subsequently, 400 μL RIPA lysate was added to each well, after which, the liquid in the well was collected and centrifuged (18,000 r/min, 10 min) to obtain the supernatant to examine the concentration of GSH, using the GSH assay kit.

### 3.8. Intracellular ROS Detection

We inoculated HeLa cells into confocal culture dishes. Subsequently, these cells were treated with (1) Control, (2) H_2_O_2_, (3) HICT, (4) HICT + H_2_O_2_, and (5) HICT + H_2_O_2_ + Laser and stained with DCFH-DA for 10 min. Finally, the fluorescence imaging was collected by CLSM.

### 3.9. Cell Viability Evaluation

Cell viability mediated by the different treatments was investigated by MTT assay. In short, HeLa, 4T1, HU-EVC, or MCF-10A cells were seeded on a 96-well plate with a density of 5 × 10^3^ cells per well for 24 h. Different concentrations of HICT were added to the cells, which were cultured for 24 h. To evaluate the phototoxicity of the HICT, the 808 nm laser (1.0 W/cm^2^, 10 min) was irradiated at 8 h after incubation, and the culturing continued for 16 h. We added 20 μL MTT (5.0 mg/mL) to each well and incubated it for 4 h. Subsequently, MTT was added for co-incubation, and the absorbance at 570 nm was recorded.

### 3.10. Live/Dead Cell Staining Assay

The inoculation method of the HeLa cells was consistent with the above. After different treatments and continuing incubated for 16 h, Calcein-AM and PI were added and incubated for 10 min. Finally, the fluorescence imaging was conducted by CLSM.

### 3.11. Animal Experiments

All animal experiments were reviewed and approved by the Animal Ethics Committee of the Institute of Process Engineering. The female nude mice (about 20 g) were purchased from the Center for Experimental Animals, Institute of Process Engineering, Chinese Academy of Science (Beijing, China). We subcutaneously injected 60 μL 4T1 cells (1 × 10^6^) in RPMI-1640 medium to establish the tumor model. The calculation formula for the tumor volume (*V*) is *V* = (*L* × *W*^2^)/2.

### 3.12. In Vivo Photothermal Imaging

The in vivo photothermal imaging was conducted 12 h post injection of HICT, followed by 808 nm laser irradiation for 10 min. We recorded the photothermal imaging using an IR camera at different irradiation times.

### 3.13. In Vivo Anticancer Effect Evaluation

We randomly divided the tumor-bearing nude mice into 4 groups: (i) Control; (ii) 808 nm laser; (iii) HICT (100 μL, 10 mg/mL); (iv) HICT (100 μL, 10 mg/mL) followed by 808 nm laser irradiation (1.0 W/cm^2^, 10 min). After the treatments, we recorded the tumor size and weight of all mice every two days.

## 4. Conclusions

In this work, a nanotheranostic platform based on biodegradable metal complex-gated organosilica was prepared for dually enhanced CDT through GSH depletions and NIR light-triggered photothermal effects. First, the hollow organosilica spheres (HOS) were initially synthesized by a “selective etching strategy”. Then, the photothermal drug IR820 was loaded into the pores of the HOS. Subsequently, a “metal complex-gated” strategy was developed to decorate the Cu-TA complex on the surface of the HOS to gate the pores completely to prevent the premature leakage of IR820, and the new nanoplatform named HICT was finally obtained. This nanoplatform had the following advantages: (1) the uniform-sized HOS can selectively degrade in TME; (2) the deposition of the Cu-TA complex blocks the pores of the HOS to effectively prevent the leakage of IR820 and remarkably increase the loading capacity; (3) the Cu-TA complex responsively degrades in the acidic TME and achieves GSH-responsive CDT; (4) the efficient NIR light-triggered photothermal effect (PCE, η = 24.7%) and GSH consumption enhance the CDT therapeutic efficacy. The in vitro and in vivo results indicate that the HICT has excellent anticancer efficacy. Comprehensively speaking, this work presented a new type of high-performance and multifunctional nanotheranostic platform for tumor treatment.

## Data Availability

Data are contained within the article and Supplementary Materials.

## Supplementary Materials

The following supporting information can be downloaded at https://www.mdpi.com/article/10.3390/molecules29051177/s1, Figure S1: TEM images of (a,b) HS@HOS and (d,e) HOS with different resolutions, along with the size distribution of (c) HS@HOS and (f) HOS by DLS, respectively; Figure S2: (a) XPS spectrum of HICT. (b–f) High-resolution XPS spectra of (b) Cu 2p, (c) Si 2p, (d) S 2p, (e) O 1s, (f) C 1s in HICT; Figure S3: (a) Nitrogen adsorption–desorption isotherms of HOS and HCT, (b) pore size distribution of HOS and HCT; Figure S4: The ability of DTNB to detect GSH consumption of HICT at different concentrations; Figure S5: Cytotoxicity of HU-EVC cells (a) and MCF-10A cells (b) after being treated by different concentrations of HOS and HICT for 24 h; Figure S6: CLSM images of cellular uptake of Rhb@HICT after 4, 8, 12 h; Figure S7: The H&E staining images of major organs after different treatments.
