# Supplementary material for: Biodegradable Metal Complex-Gated Organosilica for Dually Enhanced Chemodynamic Therapy through GSH Depletions and NIR Light-Triggered Photothermal Effects
Source: Molecules. 2024 Mar 6;29(5):1177. doi: 10.3390/molecules29051177 (PMC10933832; doi:10.3390/molecules29051177)
Supplement: Supplementary file 1 [file molecules-29-01177-s001.zip › molecules-2877882-supplementary.pdf]

Supplementary Information

Biodegradable Metal Complex-Gated Organosilica for Dually Enhanced Chemodynamic Therapy through GSH Depletions and NIR Light-Triggered Photothermal Effects

Lin Kong [1,2], Jian Li [1,2,*], Yunxiu Zhang [1,2], Jian Wang [1,2], Ke Liang [1,2], Xiaokuang Xue [1,2], Tiejin Chen [1,2], Yongliang Hao [1,2], Haohui Ren [1], Pengfei Wang [1,2] and Jiechao Ge [1,2,*]

[1] Key Laboratory of Photochemical Conversion and Optoelectronic Materials and CityU-CAS Joint Laboratory of Functional Materials and Devices, Technical Institute of Physics and Chemistry, Chinese Academy of Sciences, Beijing 100049, China; konglin20@mails.ucas.ac.cn (L.K.); zhangyunxiu114@mails.ucas.ac.cn (Y.Z.); wangjian18@mails.ucas.edu.cn (J.W.); liangke18@mails.ucas.ac.cn (K.L.); xuexiaokuang19@mails.ucas.ac.cn (X.X.); chentiejin20@mails.ucas.ac.cn (T.C.); haoyongliang22@mails.ucas.ac.cn (Y.H.); lindaroxanneren@mail.ipc.ac.cn (H.R.); wangpf@mail.ipc.ac.cn (P.W.)

[2] School of Future Technology, University of Chinese Academy of Sciences, Beijing 100049, China

* Correspondence: lijian191@mails.ucas.ac.cn (J.L.); jchge2010@mail.ipc.ac.cn (J.G.)

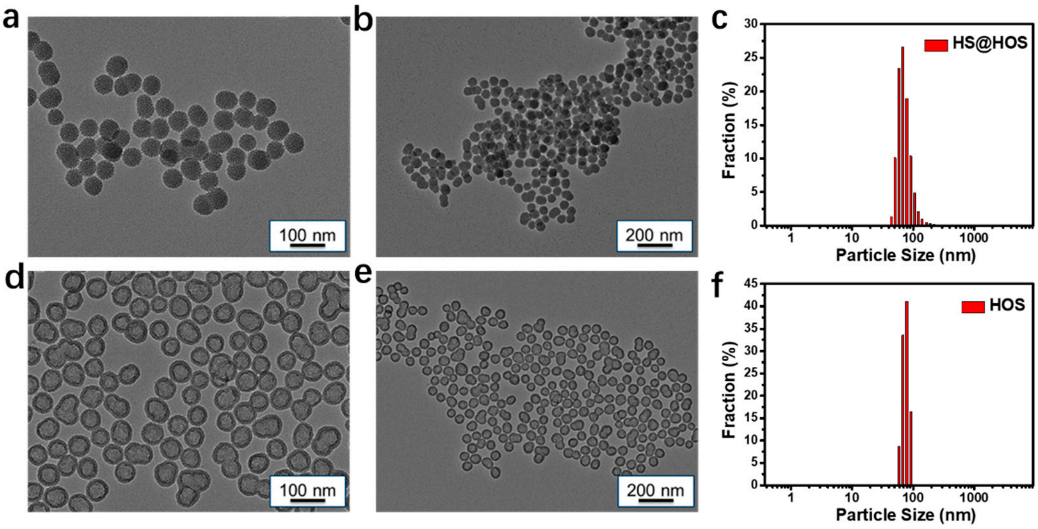

Figure S1. TEM images of (a,b) HS@HOS and (d,e) HOS with different resolution, along with the size distribution of (c) HS@HOS and (f) HOS by DLS, respectively.

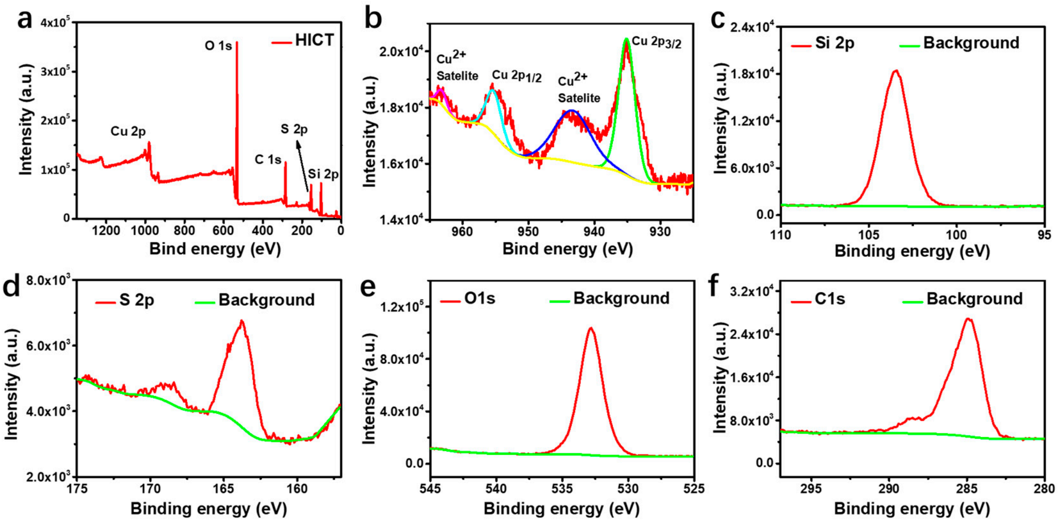

Figure S2. (a) XPS spectrum of HICT. (b-f) High-resolution XPS spectra of (b) Cu 2p, (c) Si 2p, (d) S 2p, (e) O 1s, (f) C 1s in HICT.

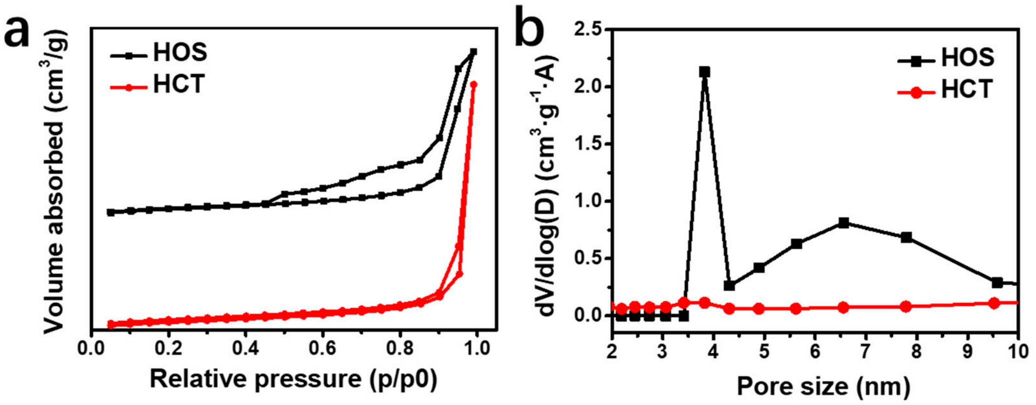

Figure S3. (a) Nitrogen adsorption-desorption isotherms of HOS and HCT, (b) Pore size distribution of HOS and HCT.

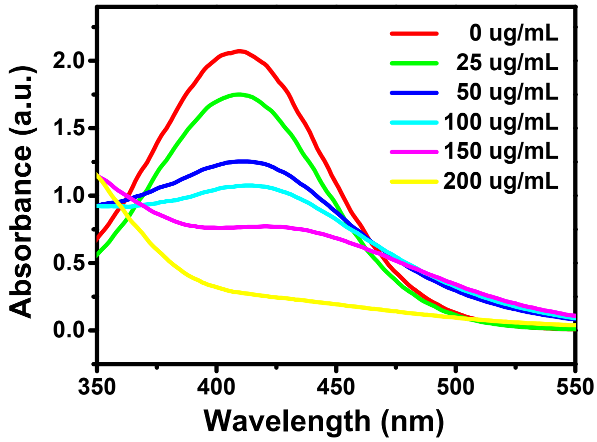

Figure S4. The ability of DTNB to detect GSH consumption of HICT at different concentrations.

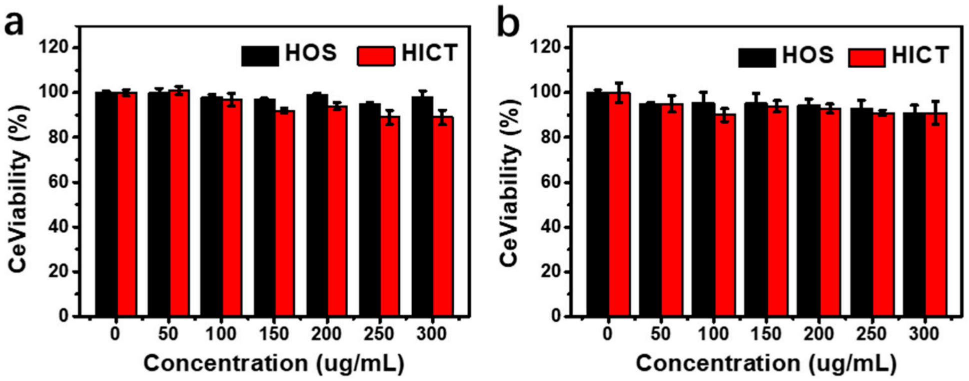

Figure S5. Cytotoxicity of HU-EVC cells (a) and MCF-10A cells (b) after being treated by different concentrations of HOS and HICT for 24 h.

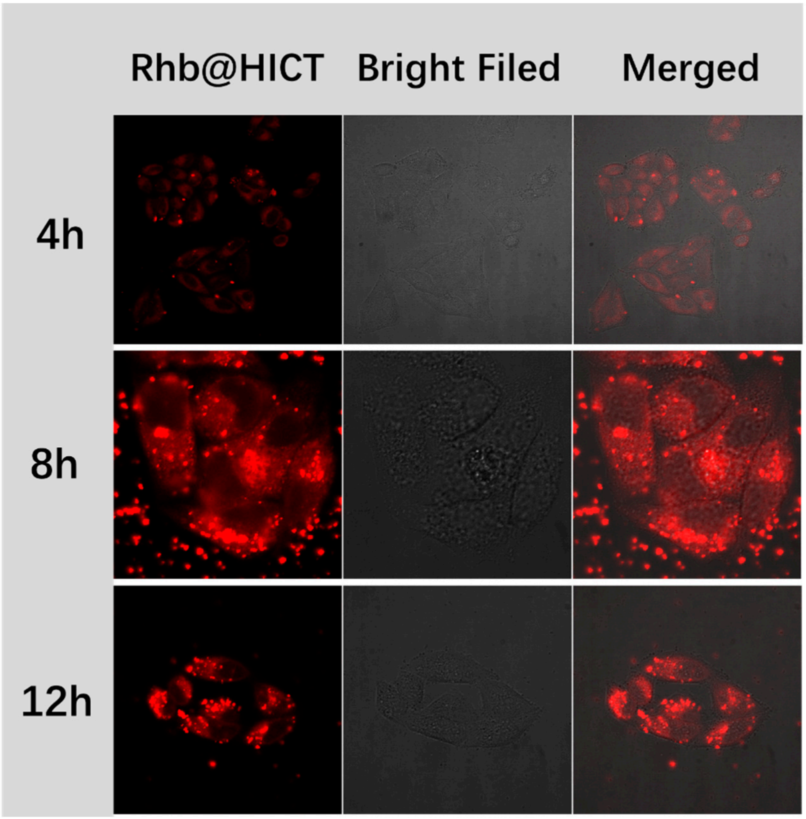

Figure S6. CLSM images of cellular uptake of Rhb@HICT after 4, 8, 12h.

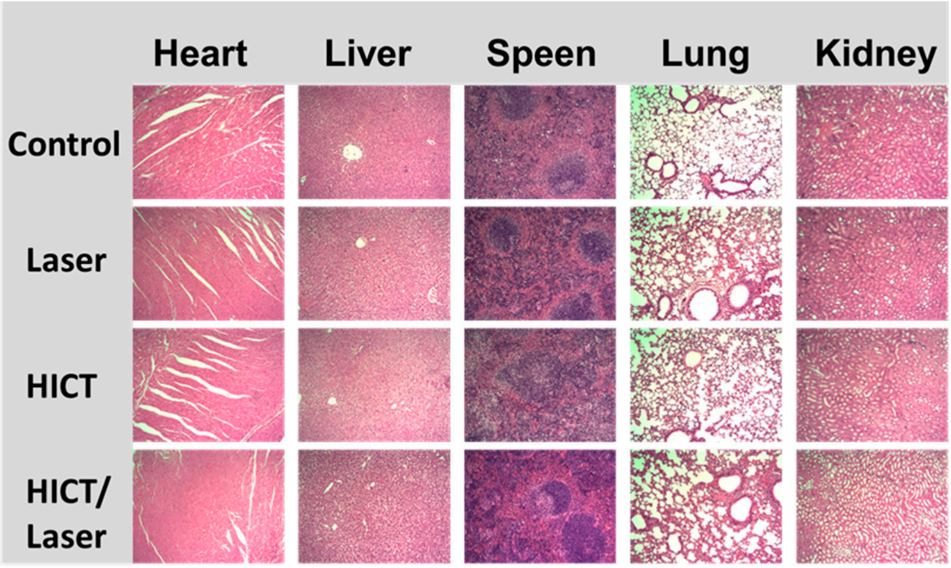

Figure S7. The H&E Staining images of major organs after different treatments.
